# Molecular mechanisms of mast cells in mediating heart failure

**DOI:** 10.3389/fimmu.2026.2003497

**Published:** 2026-10-28

**Authors:** Letao Zhou, Yifei Li, Peng Yue

**Affiliations:** Key Laboratory of Birth Defects and Related Diseases of Women and Children of Ministry of Education (MOE), Department of Pediatrics, West China Second University Hospital, Sichuan University, Sichuan, Chengdu, China

**Keywords:** cardiac remodeling, cardiomyopathy, chymase, fibrosis, heart failure, mast cells, neuroimmune signaling

## Abstract

Cardiac mast cells have emerged as mechanistically versatile and clinically consequential mediators of heart failure and cardiomyopathy, acting through a diverse arsenal of preformed granule constituents, *de novo* synthesized lipid mediators, cytokines, and chemokines that collectively orchestrate myocardial injury, adverse structural remodeling, and neurohormonal dysregulation. This narrative review examines the principal molecular and cellular mechanisms through which cardiac mast cells contribute to heart failure pathogenesis, encompassing the local renin–angiotensin–aldosterone system, extracellular matrix remodeling, chemokine-mediated immune cell recruitment, preformed mediator biology, neuroimmune crosstalk, and metabolic signaling pathways. Evidence from genetic mast cell–deficiency models, pharmacological stabilization studies, transcriptomic analyses of failing human hearts, and clinical correlative investigations supports context-dependent contributions of mast cell activation to disease pathogenesis across ischemic, inflammatory, hypertensive, metabolic, and peripartum cardiomyopathy subtypes. The review further synthesizes the evidence base for mast cell–targeted therapeutic strategies—including mast cell stabilizers, chymase inhibitors, histamine receptor antagonists, neurokinin-1 receptor antagonists, and intracellular signaling modulators—and provides an integrated perspective on their potential to complement existing neurohormonal pharmacotherapy in heart failure management. Finally, emerging frontiers including single-cell transcriptomics, glyco-epigenetic regulation, right ventricular failure, and heart failure with preserved ejection fraction are discussed as priority areas for future investigation.

## Introduction

Heart failure (HF) and cardiomyopathy collectively represent one of the most burdensome categories of cardiovascular disease worldwide, characterized by progressive myocardial dysfunction, adverse structural remodeling, and high rates of morbidity and mortality despite advances in neurohormonal pharmacotherapy. The pathophysiology of these conditions is multifactorial, involving cardiomyocyte loss, interstitial fibrosis, neurohormonal dysregulation, and chronic low-grade inflammation, yet the cellular and molecular mediators that orchestrate the transition from compensated myocardial stress to overt heart failure remain incompletely understood. Among the immune cell populations resident within the myocardium, mast cells (MCs) have emerged over the past two decades as particularly consequential effectors whose contributions to cardiac pathology extend far beyond their classical roles in allergy and anaphylaxis. MCs are long-lived, tissue-resident innate immune effectors derived from bone marrow progenitors that complete their maturation within peripheral tissues under the influence of stem cell factor (SCF) and other tissue-derived signals (1). In the heart, SCF has been shown to be responsible for the rapid expansion of mature mast cell density in response to acute myocardial stress, through maturation of resident immature cells rather than proliferation (1). Cardiac MCs are concentrated at perivascular and perineuronal niches, where they are anatomically positioned to sense and respond to hemodynamic, neurohormonal, and immunological perturbations (2, 3). This strategic localization enables mast cell–derived mediators to act in a highly spatially restricted manner, generating local concentrations of vasoactive, proteolytic, and pro-inflammatory molecules that far exceed what could be achieved through systemic delivery, and that directly impinge on cardiomyocyte contractility, vascular tone, extracellular matrix (ECM) integrity, and neurohormonal signaling in the immediate pericellular microenvironment (2).

The mediator repertoire of cardiac mast cells is extraordinarily diverse, encompassing preformed granule constituents, histamine, tryptase, chymase, cathepsin G, renin, carboxypeptidase A, and heparin, as well as *de novo* synthesized lipid mediators, cytokines, and chemokines that are generated following degranulation (3, 4). This dual-phase release program allows mast cells to exert both immediate and sustained effects on the myocardial microenvironment, with the preformed mediators providing an instantaneous effector response and the newly synthesized products shaping the subsequent inflammatory and remodeling milieu over hours to days. In the human HMC-1 mast cell line, Hara et al. demonstrated the presence and release of preformed angiotensin II (Ang II). This cell-line finding does not establish the same process in normal human cardiac mast cells (5). The importance of this local mast cell–RAS axis for cardiac pathophysiology has been substantiated by a series of mechanistic studies demonstrating that mast cell–derived renin drives local Ang II generation, norepinephrine overflow, and arrhythmogenesis during ischemia/reperfusion, a pathway whose activity is markedly reduced in mast cell–deficient hearts (6).

Existing studies have reported increased mast cell density in diseased compared with normal myocardium across several cardiomyopathy settings. However, differences in staining methods, sampled myocardial regions, and normalization procedures may limit direct comparisons across studies. Batlle et al. demonstrated a significant positive correlation between mast cell density and myocardial fibrosis in congestive heart failure patients, providing direct human evidence that mast cell accumulation is quantitatively linked to the fibrotic remodeling that drives diastolic dysfunction and ventricular stiffening (7). Akgul et al. demonstrated quantitative changes in mast cell populations following left ventricular assist device (LVAD) implantation, with mast cell numbers increasing despite reduced fibrosis during structural reverse remodeling, suggesting that the elevated mast cell density in end-stage heart failure is dynamically responsive to changes in myocardial load and injury signals rather than being a fixed pathological feature (8). These clinical observations are complemented by the comprehensive review of Poto, Marone, Galli, and Varricchi, who synthesized the breadth of evidence implicating mast cells as a novel therapeutic avenue for cardiovascular diseases, encompassing ischemic heart disease, heart failure, arrhythmias, and inflammatory cardiomyopathy, and identifying the mast cell as an underexplored but pharmacologically tractable target across the cardiovascular disease spectrum (4). The emerging prominence of cardiac mast cells as mediators of adverse myocardial remodeling was further articulated by Janicki, Brower, and Levick, who reviewed evidence from volume and pressure overload models demonstrating that mast cell activation drives the fibrogenic, inflammatory, and neurohormonal cascades that underpin the transition from adaptive to maladaptive cardiac remodeling (9).

The recognition that cardiac mast cells are not uniformly pathological but may also exert context-dependent protective functions, as demonstrated by the exacerbation of cardiac remodeling in mast cell–deficient animals under certain injury conditions (10), adds a layer of biological complexity that must be carefully considered in the design of mast cell–targeted therapeutic strategies. In this review, “context-dependent” refers to variation in mast cell effects according to disease subtype, activating stimulus, stage of injury or repair, species, and mast cell subset. This duality reflects the pleiotropic nature of the mast cell mediator repertoire and the dependence of mast cell effector polarity on the nature, intensity, and chronicity of the activating stimulus. Understanding the molecular mechanisms through which cardiac mast cells contribute to heart failure and cardiomyopathy is therefore not only of fundamental biological importance but also of direct translational relevance, given the accumulating evidence that mast cell–targeted interventions, encompassing mast cell stabilizers, protease inhibitors, receptor antagonists, and intracellular signaling modulators, can meaningfully alter disease trajectory across multiple cardiomyopathy subtypes (4, 9). The following sections of this review examine these molecular and cellular mechanisms, encompassing the renin-angiotensin-aldosterone system (RAAS), ECM remodeling, chemokine and immune cell recruitment, preformed mediator biology, neuroimmune crosstalk, and metabolic signaling pathways, with the aim of providing an integrated mechanistic framework to guide future therapeutic targeting of this clinically important cellular effector. The context-dependent disease associations and principal signaling pathways are summarized in **Figures 1** and **2**, respectively.

**Figure 1:**
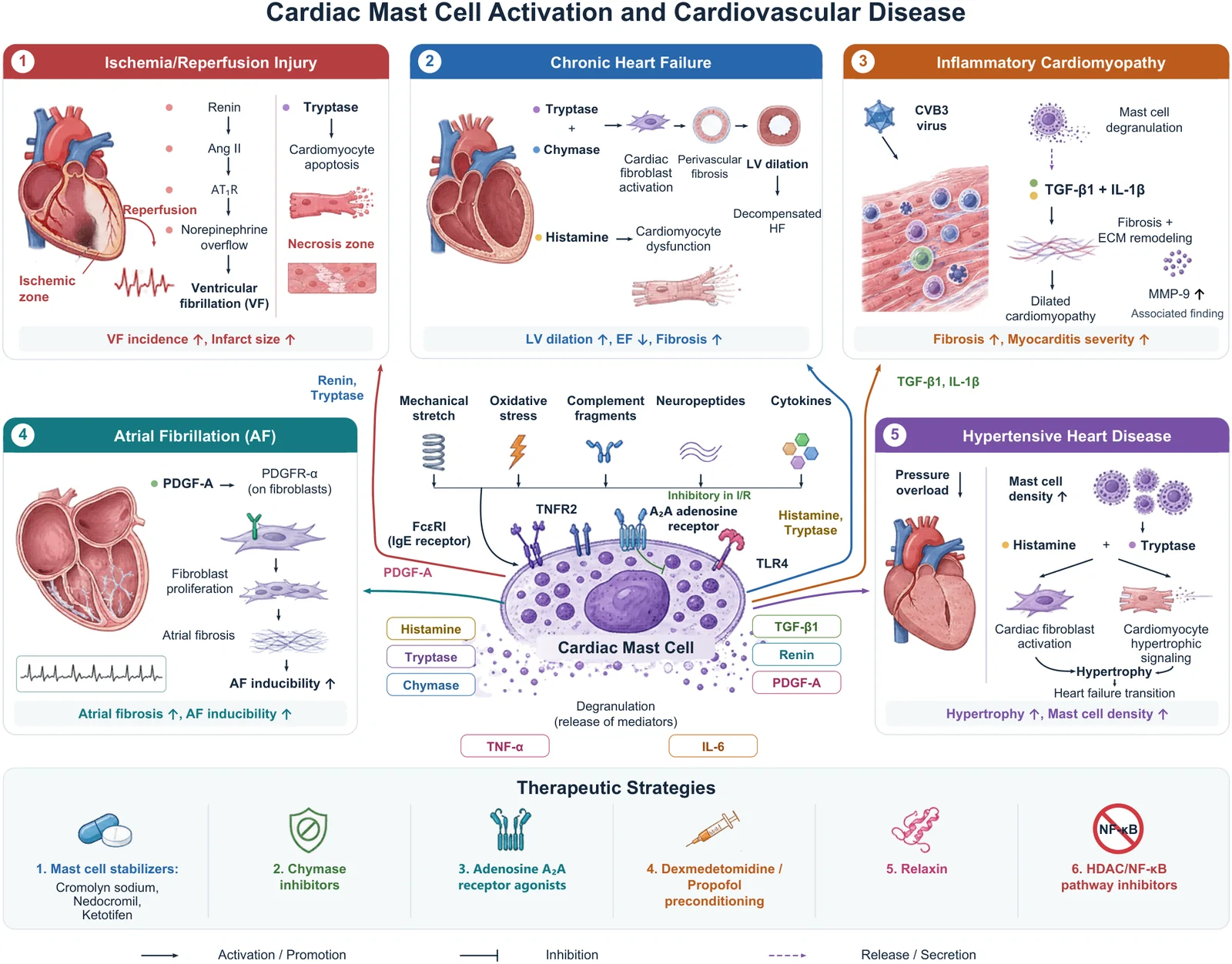
Cardiac mast cell activation as a central mediator of cardiovascular disease pathogenesis. Cardiac mast cells integrate diverse pathological stimuli, including mechanical stretch, oxidative stress, complement fragments, neuropeptides, and cytokines, triggering degranulation and release of key mediators (histamine, tryptase, chymase, renin, PDGF-A, TGF-β1, TNF-α, IL-6). Surface receptors shown include FcϵRI, TNFR2, the A_2_A adenosine receptor, and TLR4; A_2_A receptor activation can inhibit mast cell degranulation during ischemia/reperfusion (I/R). These mediators drive five distinct cardiovascular pathologies: ischemia/reperfusion injury (renin–Ang II–ventricular fibrillation axis), chronic heart failure (perivascular fibrosis and LV dilation), inflammatory cardiomyopathy (TGF-β1-driven ECM remodeling), atrial fibrillation (PDGF-A–mediated atrial fibrosis), and hypertensive heart disease (cardiomyocyte hypertrophy). Therapeutic strategies targeting mast cell stabilization and downstream mediator pathways are summarized at the bottom.

**Figure 2:**
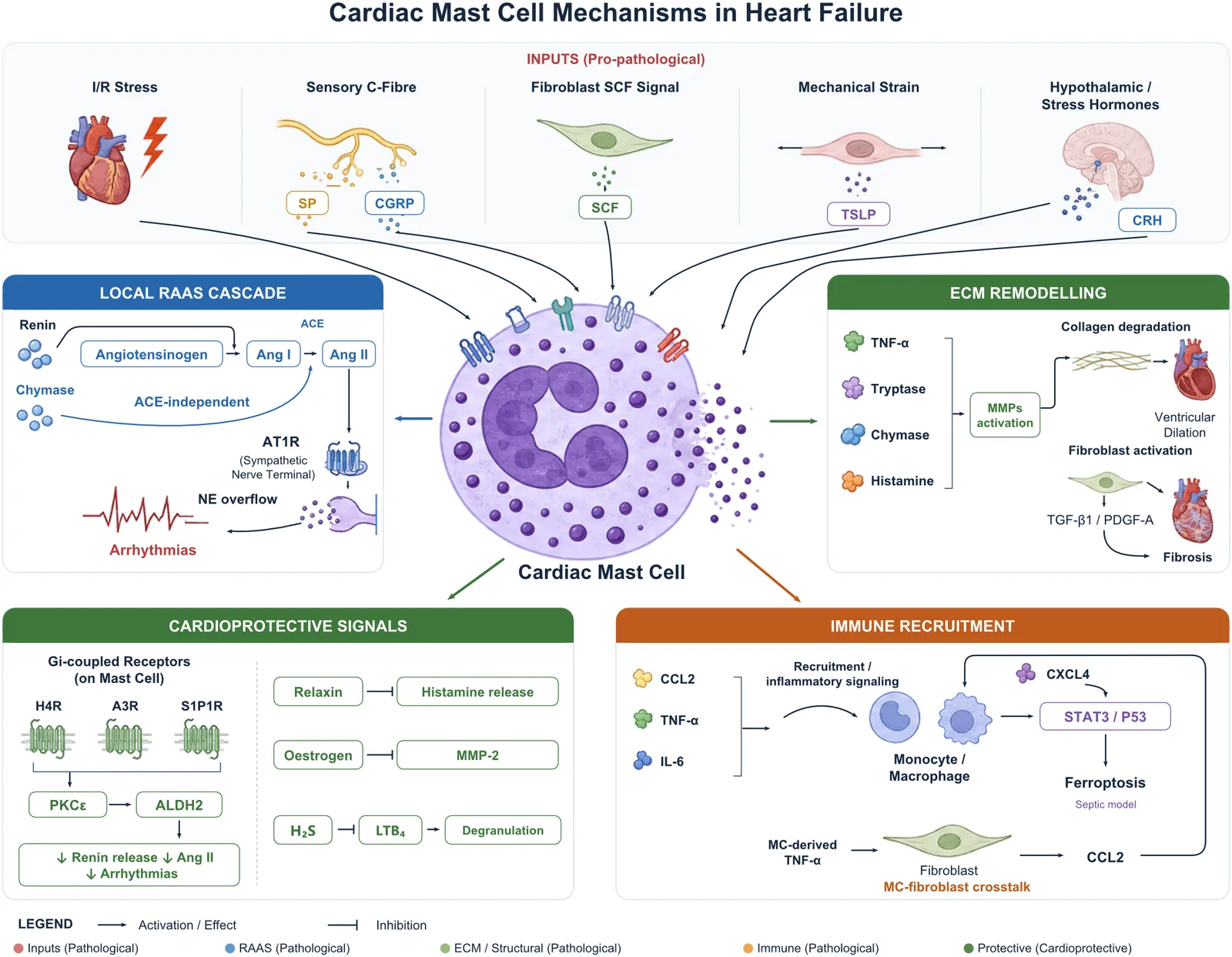
Multimechanistic roles of cardiac mast cells in heart failure pathogenesis. Cardiac mast cells serve as central integrative hubs that receive pro-pathological inputs, including ischemia/reperfusion stress, sensory C-fiber neuropeptides (SP, CGRP), fibroblast-derived SCF, mechanical strain (TSLP), and hypothalamic CRH, and transduce them into major effector programs: local RAAS activation via renin and chymase leading to Ang II generation and arrhythmias; ECM remodeling involving MMP-driven collagen degradation and fibroblast activation; immune cell recruitment and inflammatory signaling involving CCL2, TNF-α, and IL-6; and mast cell–fibroblast crosstalk, in which mast cell-derived TNF-α stimulates fibroblast CCL2 production. The reported CXCL4–STAT3/p53 pathway is linked to macrophage ferroptosis in a septic cardiomyopathy model and is shown separately. Cardioprotective signals, including the H4R/A3R/S1P1R–PKCϵ–ALDH2 axis, relaxin, estrogen, and H_2_S, counterbalance pathological degranulation. Solid arrows denote activation or effects; T-ended lines denote inhibition.

For this review, we searched Europe PMC, including PubMed-indexed records, using terms related to mast cells, heart failure, cardiomyopathy, and cardiac remodeling. Studies reporting harmful, protective, or neutral effects of mast cells were considered to provide a balanced account of their roles across different disease contexts.

## Association between mast cells and cardiovascular diseases

The association between cardiac mast cell activation and cardiovascular disease has been established across a remarkably broad spectrum of pathological conditions, ranging from acute ischemic syndromes and arrhythmias to chronic heart failure, inflammatory cardiomyopathy, atrial fibrillation, and metabolic cardiomyopathy. The breadth of this association reflects the versatility of the mast cell as a signal integrator capable of responding to mechanical stretch, oxidative stress, neuropeptides, complement fragments, immunoglobulins, and cytokines, and of translating these inputs into a coordinated effector program that shapes both the acute and chronic phases of myocardial injury and remodeling. Triggiani et al. provided an important conceptual framework by reviewing the evidence linking allergy and mast cell activation to cardiovascular pathology, highlighting the mechanistic connections between IgE-mediated mast cell degranulation, histamine release, and adverse cardiovascular outcomes including coronary vasospasm, arrhythmias, and myocardial dysfunction (11). This allergic–cardiovascular interface is clinically relevant given the high prevalence of atopic disease in the general population and the documented association between allergic conditions and increased cardiovascular risk, and it underscores the importance of considering mast cell activation as a potential contributor to cardiovascular events in susceptible individuals (11).

The most direct and mechanistically detailed evidence for a causal role of cardiac mast cells in myocardial disease was provided by Hara et al., who demonstrated in mast cell–deficient W/Wv mice subjected to aortic banding that wild-type animals progressed inexorably to decompensated heart failure with pulmonary congestion by 15 weeks, while mast cell–deficient mice maintained preserved left ventricular (LV) performance throughout, with markedly attenuated perivascular fibrosis and chymase upregulation (12). This study supported a role for mast cells in the hypertrophy-to-heart failure transition in this model and provided the conceptual impetus for a generation of mechanistic investigations into the specific molecular pathways through which cardiac mast cell activation drives myocardial dysfunction. However, Kit mutations also affect other cell populations, limiting mast cell–specific causal inference (13). This limitation applies throughout this review to findings from Kit-dependent mast cell-deficient models. The question of whether cardiac mast cells are “innocent bystanders or key actors” in heart failure, posed by Palladini et al. in a seminal editorial, has since been supported by experimental studies, although human correlative findings alone do not establish causality (14). Balakumar et al. synthesized data from hypertension, pressure overload, and neurohormonal activation models to demonstrate that resident cardiac mast cell density has been reported to increase in experimental cardiac hypertrophy, and that mast cell stabilization attenuates hypertrophic responses, implicating the full mediator repertoire of cardiac mast cells, histamine, tryptase, chymase, and cytokines, in the activation of cardiac fibroblasts and the promotion of hypertrophic signaling in cardiomyocytes (15). Kolck et al. further emphasized the clinical implications of cardiac mast cell activation for heart failure pathophysiology, highlighting the strategic perivascular and perineuronal localization of cardiac mast cells as the anatomical basis for their capacity to rapidly modulate cardiac function through vasoactive mediator release (2).

In the context of acute ischemia/reperfusion (I/R) injury, Mackins et al. showed that mast cell degranulation at reperfusion releases renin, which activates local Ang II formation, AT1 receptor–mediated norepinephrine overflow, and ventricular fibrillation, with mast cell–deficient hearts exhibiting substantially reduced reperfusion arrhythmic activity relative to wild-type controls (6). Rork et al. showed that the adenosine A_2_A receptor (A_2_AAR) agonists ATL146e and CGS-21680 reduced infarct size from 38 ± 2% of the area at risk to 24 ± 2% and 22 ± 2%, respectively, and decreased tryptase release in isolated perfused mouse hearts. These effects were absent in A_2_AAR-deficient hearts. Mast cell-deficient hearts had a lower baseline infarct size, and ATL146e provided no significant additional protection. These findings support a contribution of reduced mast cell degranulation to A_2_AAR-mediated cardioprotection in this model (16). Bhattacharya et al. provided additional genetic evidence by demonstrating that mast cell–deficient W/Wv mice have lower serum IL-6 and less cardiac tissue necrosis than wild-type littermates following myocardial I/R, further confirming the pro-inflammatory and pro-necrotic contributions of cardiac mast cells to reperfusion injury (17). The pharmacological tractability of mast cell stabilization as a cardioprotective strategy was validated across multiple experimental platforms: Jaggi et al. demonstrated that ketotifen and low-dose carvedilol both stabilized cardiac mast cells and reduced myocardial injury markers including LDH, CK, and infarct size in Langendorff rat hearts (18); Li et al. demonstrated that propofol pretreatment attenuates mast cell degranulation through inhibition of STIM1/Orai1-mediated store-operated calcium influx, reducing tryptase activity and cardiomyocyte apoptosis (19); Xiong et al. demonstrated that dexmedetomidine preconditioning suppresses mast cell degranulation and downstream HMGB1–TLR4–NF-κB inflammatory signaling to reduce arrhythmia incidence and infarct size (20); and the same group showed that oxytocin pretreatment stabilizes cardiac mast cells and attenuates hemodynamic disorders, arrhythmias, and NF-κB activation in rat I/R models (21). Davani et al. further refined this understanding by demonstrating through intramyocardial microdialysis that reperfusion histamine release kinetics are temporally correlated with arrhythmia severity, and that ischemic preconditioning shifts the timing of mast cell degranulation to reduce total arrhythmia burden (22). Nistri et al. demonstrated in a porcine MI model that relaxin confers cardioprotection partly through mast cell stabilization, reducing plasma histamine and the incidence of severe ventricular arrhythmias (23).

The association between cardiac mast cells and chronic heart failure has been substantiated by both experimental and clinical evidence. In volume overload heart failure, Brower and Janicki demonstrated that pharmacological mast cell stabilization with nedocromil dose-dependently prevented left ventricular dilation and contractility decline in a rat aortocaval fistula model, significantly reducing congestive heart failure morbidity and mortality, establishing that mast cell activation drives ventricular dilation in chronic volume overload (24). In the failing human heart, Akgul et al. demonstrated that mast cell properties and myocardial structural changes differ between dilated and ischemic cardiomyopathy under mechanical circulatory support, suggesting disease-specific patterns of mast cell activation and mediator release that may have implications for targeted therapy (25). The role of mast cell–derived tryptase in amplifying inflammatory signaling in the failing myocardium was demonstrated by Kinoshita et al., who showed that tryptase in mast cell granules enhances MCP-1 and IL-8 production in human endothelial cells, suggesting a mechanism for leukocyte recruitment, without directly establishing persistent inflammation in patients with end-stage heart failure (26). The pathogenic role of cardiac mast cell activation in drug-related cardiac fatalities was demonstrated by Perskvist et al., who showed that mast cell activation/degranulation, TNF-α release, and cell death are pathogenic features of acute drug-related cardiac fatalities, extending the cardiovascular relevance of mast cell activation beyond classical heart failure etiologies (27).

In inflammatory cardiomyopathy, the association between cardiac mast cells and disease severity is particularly well established. Higuchi et al. demonstrated that mast cell proteases and histamine H_1_ receptor activation are critical pathogenic mechanisms in viral myocarditis, with mast cell–deficient mice showing markedly attenuated disease and the H_1_ antagonist bepotastine improving survival (28). Mina et al. demonstrated that cromolyn sodium–mediated mast cell inhibition prevented left ventricular dilation, attenuated dysfunction, and reduced myocardial fibrosis in fulminant autoimmune myocarditis (29). The fibrogenic role of cardiac mast cells in postmyocarditis dilated cardiomyopathy was directly established by Palaniyandi Selvaraj et al., who demonstrated a significant positive correlation between mast cell density and myocardial fibrosis, and showed that disodium cromoglycate (DSCG) reduced fibrosis, mast cell infiltration, TGF-β1, and collagen III (30). Complementing these findings, Fairweather et al. demonstrated that IFN-γ deficiency dramatically exacerbated chronic CVB3-induced myocarditis, pericarditis, and fibrosis through unrestrained mast cell degranulation and elevated TGF-β1, IL-1β, and IL-4 (31), while early mast cell degranulation within six hours of CVB3 infection was identified as a determinant of long-term autoimmune heart disease susceptibility in genetically susceptible BALB/c mice (32). The gene expression of mast cell chymase and tryptase was shown by Kitaura-Inenaga et al. to increase progressively during encephalomyocarditis virus myocarditis, correlating with MMP-9 upregulation and ECM remodeling (33). The influence of environmental endocrine disruptors on cardiac mast cell activation was demonstrated by Bruno et al., who showed that bisphenol A (BPA) exposure disrupts estrogen receptor signaling to activate cardiac mast cells and T cells, exacerbating perimyocarditis and fibrosis after CVB3 infection (34).

The association between cardiac mast cells and atrial fibrillation (AF) represents a particularly clinically significant dimension of mast cell cardiovascular biology. Liao et al. established in a pressure overload mouse model that cardiac mast cells drive AF through PDGF-A–mediated fibroblast proliferation and atrial fibrosis via PDGFR-α signaling, with mast cell stabilization or deficiency attenuating both atrial fibrosis and AF inducibility (35). The role of cardiac mast cells in the failing human heart has been further characterized through studies of patients undergoing mechanical circulatory support. Akgul et al. demonstrated that LVAD implantation is associated with increased mast cell density despite reduced fibrosis during structural reverse remodeling (8), while Akgul, Skrabal, and colleagues characterized the mediator profiles of mast cells in explanted failing hearts, providing insight into the specific effector pathways active in end-stage disease (36). Palaniyandi et al. demonstrated that chymase inhibition reduces the progression to heart failure after autoimmune myocarditis in rats, further establishing the mast cell chymase pathway as a therapeutic target in inflammatory cardiomyopathy (37). The context-dependent nature of cardiac mast cell function is illustrated by the finding of Joseph et al. that mast cell deficiency exacerbated hyperhomocysteinemia-induced cardiac remodeling and diastolic dysfunction in Ws/Ws rats, revealing a protective mast cell function in this metabolic injury context (10). Taken together, the association between cardiac mast cells and cardiovascular disease is now established across ischemic heart disease, chronic heart failure, inflammatory cardiomyopathy, atrial fibrillation, hypertensive heart disease, and metabolic cardiomyopathy, with consistent evidence from genetic mast cell–deficiency models, pharmacological stabilization studies, and clinical correlative analyses that mast cell activation is a mechanistically active contributor to disease pathogenesis rather than an epiphenomenon of myocardial injury.

## Mast cells and the local renin–angiotensin–aldosterone system

### Cardiac mast cells as a cellular source of renin and initiators of local angiotensin II formation

The renin–angiotensin–aldosterone system (RAAS) has long been recognized as a central neurohormonal axis governing cardiac remodeling and the progression of heart failure. Whereas circulating RAAS components have been extensively characterized, a growing body of evidence has established that cardiac mast cells constitute a critical cellular reservoir for renin within the myocardium itself, thereby initiating a spatially restricted, autocrine–paracrine angiotensin II (Ang II) cascade that operates largely independently of systemic RAAS activation. Seminal work by Mackins et al. demonstrated that ischemia/reperfusion (I/R) triggers the degranulation of resident cardiac mast cells, releasing renin that locally converts angiotensinogen to angiotensin I and subsequently to Ang II (6). Using guinea pig and mouse isolated heart I/R models, the authors showed that the selective renin inhibitor BILA2157 abolished renin-dependent Ang I–forming activity and markedly attenuated both norepinephrine (NE) release and ventricular fibrillation, while hearts from mast cell–deficient mice exhibited substantially less arrhythmic activity than wild-type controls (6). These findings placed cardiac mast cell–derived renin at the apex of a pathological signaling cascade that culminates in sympathetic activation and life-threatening arrhythmias. Complementary clinical evidence was provided by Batlle et al., who examined myocardial biopsies from 30 end-stage heart failure patients and demonstrated that both ACE and chymase mRNA expression were significantly elevated in failing myocardium, accompanied by a marked increase in mast cell density relative to non-failing controls (38). Notably, ACE2 expression was unchanged while eNOS was reduced, suggesting a net shift toward vasoconstriction and pro-fibrotic signaling driven in part by mast cell–associated proteases (38).

### Neuroimmune amplification: sensory C-fiber–mast cell crosstalk in local RAS activation

The local RAAS cascade initiated by cardiac mast cell renin is substantially amplified through bidirectional interactions with the cardiac sensory nervous system. Morrey et al. characterized this neuroimmune axis in guinea pig isolated hearts by demonstrating that capsaicin-induced activation of sensory C-fibers releases substance P (SP) and calcitonin gene-related peptide (CGRP), which in turn trigger mast cell degranulation and renin secretion (39). The resulting local Ang II formation stimulated AT1 receptors on sympathetic nerve terminals, eliciting NE overflow and arrhythmic dysfunction; selective SP receptor blockade or pharmacological mast cell stabilization attenuated all of these downstream effects (39). The therapeutic implications of disrupting this circuit were further explored by Reid et al. in a comprehensive pharmacological review, which proposed that a multi-target strategy, encompassing mast cell stabilization, renin inhibition, local ACE blockade, AT1 receptor antagonism, and histamine H3 receptor activation, could target additional pathways beyond ACE inhibition, without establishing superior clinical outcomes in heart failure (40). The same group subsequently reviewed the broader significance of renin storage within cardiac mast cells, noting that mast cell–derived renin initiates not only local angiotensin formation but also downstream consequences including coronary vasoconstriction, cardiomyocyte apoptosis, fibroblast proliferation, and endothelin release, a repertoire of effects that collectively accelerate the transition from compensated hypertrophy to overt heart failure (41).

### Mast cell chymase: an ACE-independent pathway of cardiac angiotensin II generation

Cardiac mast cells also release chymase, a serine protease capable of generating Ang II directly from angiotensin I in a manner entirely independent of ACE. This alternative pathway may limit suppression of myocardial Ang II by ACE inhibition in experimental models. Wei et al. provided mechanistic evidence for this limitation in mice and hamsters by demonstrating that chronic ACE inhibition paradoxically upregulated LV chymase activity through a bradykinin/B2 receptor–mediated mechanism, thereby maintaining elevated interstitial Ang II concentrations despite pharmacological ACE blockade. The study also showed that combined chymase and ACE inhibition significantly improved LV function, reduced adverse remodeling, and improved post-MI survival compared with ACE inhibition alone (42). Matsumoto et al. demonstrated in a dog tachycardia-induced heart failure model that chymase inhibition reduced mast cell density and cardiac Ang II levels, decreased LV end-diastolic pressure, shortened the time constant of isovolumic relaxation (tau), and suppressed collagen I/III and TGF-β mRNA expression, establishing chymase-mediated Ang II generation as a critical driver of both diastolic dysfunction and interstitial fibrosis (43). Kokkonen et al. further contextualized these observations by reviewing chymase’s dual capacity to generate Ang II and to directly activate TGF-β, process collagen precursors, and degrade other regulatory substrates, concluding that chymase operates through both Ang II–dependent and –independent mechanisms simultaneously (44).

### Dynamic regulation of cardiac mast cell populations by the RAAS and hydrogen sulfide

The relationship between cardiac mast cells and the RAAS is bidirectional: RAAS activity itself modulates mast cell density and phenotype in a compartment-specific and temporally dynamic manner. Erokhina et al. tracked mast cell population dynamics in a rat isoproterenol-induced heart failure model and found that pericardial mast cell density increased 1.5-fold at 24 hours and 14 days post-injection, rising to 2-fold by 28 days, with changes in pericardial mast cell numbers correlating significantly with functional indices of heart failure severity (45). In a subsequent study, the same group demonstrated that different RAS inhibitors differentially regulated mast cell density across cardiac compartments, implying that cardiac mast cells function as integral tissue components of the local RAAS rather than passive bystanders (46). Beyond classical pharmacological interventions, Liu et al. demonstrated that hydrogen sulfide (H_2_S), administered as the donor compound NaHS, reversed collagen deposition and renin elevation by reducing the number of degranulated cardiac mast cells through downregulation of leukotriene A4 hydrolase (LTA4H) and leukotriene B4 (LTB4), positioning H_2_S-based therapies as mechanistically distinct approaches to interrupting the mast cell–renin–Ang II cascade in heart failure (47).

## Mast cell–mediated extracellular matrix remodeling

### Mast cell–derived mediators and the bidirectional regulation of myocardial ECM homeostasis

The extracellular matrix (ECM) of the myocardium is a dynamically regulated network whose integrity is indispensable for the maintenance of ventricular geometry, cardiomyocyte alignment, and contractile force transmission. Disruption of ECM homeostasis, whether through excessive fibrillar collagen degradation leading to ventricular dilation or through pathological collagen deposition culminating in interstitial fibrosis, constitutes a central structural determinant of heart failure progression. Cardiac mast cells are pivotal regulators of both arms of this remodeling process, acting through a diverse arsenal of preformed and newly synthesized mediators that converge on the matrix metalloproteinase (MMP) system and the fibroblast compartment (48, 49). Janicki et al. synthesized data from pressure and volume overload animal models alongside human cardiomyopathy specimens to demonstrate that mast cell–derived TNF-α, tryptase, and chymase collectively activate MMPs to degrade fibrillar collagen, thereby precipitating ventricular dilation (48). Levick et al. provided an integrative synthesis demonstrating that mast cell–derived histamine, chymase, tryptase, and TNF-α act in concert on fibroblasts, endothelial cells, smooth muscle cells, and cardiomyocytes to activate MMPs and promote collagen turnover in a hemodynamic load–dependent manner, with volume-overloaded hearts exhibiting net collagen degradation and chamber dilation while pressure-overloaded hearts show net collagen deposition and fibrosis (49). Direct clinical corroboration was provided by Batlle et al., who demonstrated that collagen fraction was three-fold higher in failing myocardium, with mast cell density exhibiting a statistically significant linear correlation with collagen fraction (R^2^ = 0.28, P < 0.005) (7). Extending these observations to hypertensive heart disease, Shiota et al. conducted serial cardiac analysis in spontaneously hypertensive rats and found that mast cells were detectable from birth, with elevated c-kit/SCF, TNF-α, NF-κB, and IL-6 expression already evident in the prehypertensive phase, and that activated mast cells became the predominant myocardial source of TGF-β1 and basic fibroblast growth factor (bFGF) as hypertrophy advanced toward overt heart failure (50).

### Mast cell–fibroblast crosstalk: TGF-β1, PDGF-A, and TSLP as intercellular fibrogenic signals

The most potent fibrogenic effects of cardiac mast cells are mediated through direct cellular crosstalk with cardiac fibroblasts. Zhang et al. provided mechanistic evidence using MHCsTNF transgenic mice crossed with mast cell–deficient *Kit^–^/^–^* mice, demonstrating that the 11-fold increase in collagen volume fraction observed in MHCsTNF mice was completely abrogated by mast cell deficiency. Mast cell–fibroblast co-culture experiments showed that mast cells enhanced fibroblast α-SMA expression, proliferation, and collagen mRNA in a manner fully blocked by TGF-β receptor I antagonism (51). Liao et al. demonstrated that pressure overload induces cardiac mast cell infiltration and upregulation of PDGF-A synthesis, which drives fibroblast proliferation and collagen expression via PDGFR-α in the atrial myocardium, with PDGFR-α neutralization reducing atrial fibrosis and substantially attenuating atrial fibrillation inducibility (35). More recently, Pimpalwar et al. identified thymic stromal lymphopoietin (TSLP) as a novel mechanosensory signal mediating fibroblast-to-mast cell communication, demonstrating that mechanical strain elevated TSLP in fibroblasts, that TSLP receptor was expressed on myocardial mast cells, and that TSLP overexpression induced mast cell TGF-β expression, constituting a feedforward loop in which mechanical stress amplifies fibrogenic mast cell activation (52). Hans et al. demonstrated in ApoE^–^/^–^ mice fed a high-fat diet that PARP-1 drives a cascade of MMP activation and reduced TIMP-2 expression, and mast cell degranulation that precipitates cardiac hypertrophy and dilation, with PARP-1 deletion attenuating these changes; the experiment did not establish a mast cell–specific causal sequence (53).

### Sex hormone modulation and mechanical unloading as regulators of mast cell–ECM interactions

Estrogen suppresses mast cell–driven ECM degradation: Chancey et al. demonstrated that compound 48/80–induced mast cell degranulation was associated with active MMP-2 levels 133% higher in ovariectomized than in intact females, together with significant collagen reduction in ovariectomized females, whereas intact and estradiol-replaced females showed no significant differences from one another in cardiac function, MMP-2 activity, or collagen volume fraction (54). Lu et al. further showed that ovariectomized females subjected to volume overload developed significant increases in mast cell density, MMP-2 activity, collagen degradation, endothelin-1, SCF, and TNF-α, whereas intact females were largely protected and nedocromil prevented these early changes in ovariectomized females, demonstrating that estrogen’s cardioprotection against volume overload is mediated in substantial part through alteration of mast cell phenotype (55). Li et al. demonstrated in a rat aortic constriction model that estrogen significantly inhibited mast cell chymase release in pressure-overloaded hearts, attenuating adverse cardiac remodeling and preserving ventricular function (56). In the clinical context of end-stage heart failure, Akgul et al. provided evidence that long-term LVAD support exceeding 40 days induced a secondary increase in mast cell numbers characterized by a phenotypic shift toward chymase-negative cells, coinciding with decreased bFGF expression and reduced interstitial fibrosis, suggesting that mechanical unloading fundamentally alters mast cell functional state rather than simply eliminating mast cells (36).

## Chemokine networks and immune cell recruitment

### Mast cell–derived chemokines and the recruitment of monocytes and macrophages in inflammatory cardiomyopathy

Cardiac mast cells function as sentinel immunological nodes that, upon activation, orchestrate the recruitment and phenotypic polarization of circulating immune cells through the release of a coordinated chemokine network. The SCF/mast cell/CCL2/monocyte/macrophage axis was systematically delineated by Luo et al. using CVB3-infected wild-type and mast cell–deficient Kit W-sh mice, demonstrating that mast cell deficiency substantially reduced myocarditis severity, cardiac fibrosis, and the influx of Ly6C^high^ monocytes and macrophages into the infected myocardium (57). Mechanistically, fibroblast-derived SCF triggered mast cell degranulation and the secretion of CCL2 and TNF-α. In turn, mast cell–derived TNF-α stimulated fibroblast expression of CCL2, α-SMA, collagen, and TGF-β. This constitutes a self-amplifying circuit through which a localized viral trigger is translated into sustained myocardial inflammation and progressive fibrosis (57). Li et al. independently established that acute volume overload was accompanied by a rapid doubling of cardiac mast cell density driven by a SCF–chymase paracrine amplification circuit, with myocardial SCF levels increasing rapidly following fistula creation and mast cell degranulation itself elevating SCF through chymase-mediated release, reflecting maturation of resident immature mast cells rather than proliferation (1). Bhattacharya et al. demonstrated that mast cell–deficient W/Wv mice exhibited significantly higher cardiac muscle viability than wild-type controls after I/R, accompanied by markedly attenuated serum IL-6 responses, supporting an association with reduced systemic inflammation, without identifying mast cells as the direct source of the measured IL-6 (17).

### Mast cell–cytokine axes in diabetic and septic cardiomyopathy: IL-6, TNF-α, and CXCL4 as disease-specific effectors

In diabetic cardiomyopathy, He et al. demonstrated that mast cell deficiency in streptozotocin-treated mice protected against diabetic cardiomyopathy, with mast cell–deficient animals showing preserved cardiac function, reduced myocardial fibrosis, attenuated macrophage infiltration, and lower TNF-α, IL-6, and TGF-β1 levels compared to diabetic wild-type controls (58). In the distinct pathophysiological context of septic cardiomyopathy, Wei et al. identified a previously unrecognized mechanism by which mast cell activation drives macrophage ferroptosis through the chemokine CXCL4, demonstrating that mast cell degranulation increased tryptase and CXCL4 expression in the myocardium, and that CXCL4 activated STAT3/P53 signaling in macrophages, inducing iron accumulation, ROS overproduction, and ferroptotic cell death, thereby impairing macrophage phagocytic capacity and exacerbating myocardial injury in the experimental LPS model (59).

### Innate immune sensing, environmental modulation, and mast cell–TLR4 cooperation in cardiac inflammatory disease

The capacity of cardiac mast cells to orchestrate immune cell recruitment is substantially shaped by innate immune pattern recognition pathways. Fairweather and Frisancho-Kiss provided a comprehensive synthesis demonstrating that mast cells regulate cardiac inflammation and fibrosis through TLR4 signaling, antigen presentation, and pro-inflammatory cytokine production, and that many existing cardiovascular drugs, including statins, beta-blockers, and ACE inhibitors, exert part of their benefit through downstream suppression of mast cell–mediated inflammatory pathways (3). The cooperative relationship between cardiac mast cells and TLR4 was directly demonstrated by Li et al. in CVB3-induced viral myocarditis, in which both mast cell numbers and TLR4 mRNA expression were significantly elevated at all serial sacrifice time points, with a strong positive correlation between mast cell count and TLR4 expression (R^2^ = 0.877), demonstrating an association but not a mutually reinforcing causal circuit (60). Bruno et al. demonstrated that BPA exposure significantly increased myocarditis severity, pericarditis, and pericardial mast cell degranulation in CVB3-infected mice, while activating estrogen receptors in the spleen and increasing cardiac fibrosis, revealing that BPA disrupts the estrogen receptor balance that normally constrains cardiac mast cell activation (34).

## Preformed mediators: histamine, proteases, and their molecular targets

### Histamine and protease-mediated disruption of cardiac contractility and inflammatory amplification

Cardiac mast cells store a diverse arsenal of preformed mediators within their secretory granules that are released instantaneously upon degranulation. Among these preformed constituents, histamine and the serine proteases tryptase, chymase, and cathepsin G are quantitatively dominant and mechanistically the most consequential in the context of heart failure and cardiomyopathy. Francis and Tang were among the first to formally propose that mast cell–derived histamine contributes to cardiac dysfunction in heart failure through histamine receptor signaling and indirect modulation of sympathetic tone and vascular resistance, raising the possibility that antihistamines might serve as adjunctive therapies in patients with decompensated heart failure (61). However, H_1_ and H_2_ effects should be distinguished: isolated human atrial muscle experiments identified H_1_-mediated negative and H_2_-mediated positive inotropic components (62). The pathogenic role of mast cell proteases in inflammatory cardiomyopathy was directly demonstrated in a study by Higuchi et al., in which mast cell protease expression was progressively upregulated from the acute to the subacute phase of infection and correlated tightly with MMP-9 upregulation, myocardial necrosis, and fibrosis, with treatment with the H_1_ receptor antagonist bepotastine improving survival and reducing cardiac injury in wild-type mice (28). Beyond their inflammatory roles, mast cell–derived tryptase exerts direct effects on cardiomyocyte contractile machinery through protease-activated receptor-2 (PAR-2) signaling: Ngkelo et al. demonstrated that mast cell tryptase normally restrains PKA activity through PAR-2 proteolysis, thereby maintaining appropriate myofilament Ca^2+^ sensitivity and contractile force generation in the post-ischemic myocardium, revealing that mast cell–derived proteases are not exclusively injurious but also serve an acute reparative function (13). Somasundaram et al. demonstrated in a canine MI model that tryptase, acting as a PAR-2 agonist on endothelial cells, selectively induced angiogenic chemokines CCL2/MCP-1 and CXCL8/IL-8 while sparing the angiostatic chemokine CXCL10/IP-10, suggesting a potential contribution to infarct neovascularization during healing (63).

### Chymase as a pleiotropic protease: from IGF-1 degradation and cardiomyocyte death to diagnostic biomarker potential

Chymase, the most abundant serine protease stored in human cardiac mast cell granules, exerts a remarkably broad spectrum of pathological effects in the failing heart. Tejada et al. showed that mouse mast cell protease-4 (MMCP-4) degrades insulin-like growth factor-1 (IGF-1) in the post-MI myocardium, thereby abolishing IGF-1 receptor survival signaling and precipitating cardiomyocyte death, with genetic deletion of *Mcpt4* markedly reducing late infarct size, preserving IGF-1R signaling, diminishing cardiomyocyte apoptosis, and attenuating adverse structural remodeling (64). Oyamada et al. reported that, in a porcine cardiac I/R model, chymase inhibition significantly reduced infarct size, troponin T release, MMP-9 activation, apoptotic cell numbers, and neutrophil infiltration, while simultaneously increasing phospho-eNOS and total eNOS expression (65). Hooshdaran et al. demonstrated that dual inhibition of cathepsin G and chymase decreased immune cell infiltration, reduced cardiomyocyte apoptosis and fibroblast activation, attenuated collagen deposition, and preserved ventricular function compared with vehicle treatment (66). Jin et al. advanced the clinical translation of chymase inhibition by developing a chymase-specific RNA aptamer (HA28) that significantly improved LVEF, fractional shortening, and E/A ratio, markedly suppressed cardiac chymase activity, and substantially reduced mortality compared to controls in a rat MI model (67). The diagnostic potential of chymase was illuminated by Mulvey et al., who demonstrated through cardiac tissue proteomics and single-nucleus RNA sequencing that chymase and carboxypeptidase A3 were consistently upregulated in peripartum cardiomyopathy hearts. Serum chymase was also reported to be strongly predictive of cardiomyopathy in peripartum women, identifying it as a diagnostic candidate requiring further clinical validation (68).

### Cathepsin G, tryptase biomarker limitations, and the therapeutic landscape of chymase inhibition

Cathepsin G–positive mast cells were not detected in normal-heart controls but constituted 46% of the mast cell population in end-stage cardiomyopathy biopsies, falling to 11% following long-term LVAD support, coinciding with reductions in myocyte necrosis, hypertrophy, and fibrosis, as demonstrated by Jahanyar et al. (69). In contrast to the tissue-level evidence for mast cell protease activity in heart failure, Upadhya et al. demonstrated that systemic serum tryptase levels do not reliably reflect myocardial mast cell activation in patients with left ventricular dysfunction, being paradoxically lower in patients with increased left ventricular end-diastolic volume and congestive heart failure, highlighting the need for tissue-based or imaging biomarkers to accurately assess mast cell activity in the failing heart (70). Doggrell and Wanstall comprehensively reviewed chymase’s pathophysiological repertoire, encompassing Ang II generation, TGF-β1 and IL-1β activation, endothelin formation, lipid metabolism alteration, and ECM degradation, and proposed orally active chymase inhibitors as promising agents for cardiac diseases in which ACE inhibitor therapy alone is insufficient (71, 72).

## Neuroimmune crosstalk and cardiac mast cell activation

The cardiac mast cell is embedded within a rich neuroimmune network in which neuropeptides, autonomic neurotransmitters, and hypothalamic stress hormones converge on mast cell surface receptors to orchestrate a sustained program of mediator release that amplifies myocardial injury and remodeling. A foundational mechanistic insight into this axis was provided by Meléndez et al., who demonstrated in a rat aortocaval fistula volume overload model and in TAC1-deficient (substance P-null) mice that substance P (SP) drives adverse cardiac remodeling through neurokinin-1 receptor (NK-1R)–mediated mast cell degranulation, with SP stimulation via NK-1R increasing cardiac mast cell density, elevating TNF-α levels, and activating MMPs, while pharmacological NK-1R antagonism with L732138 prevented all of these changes and attenuated ventricular remodeling (73). The complexity of SP’s role was subsequently refined by Widiapradja et al., who demonstrated in spontaneously hypertensive rats and angiotensin II–infused mice, including mast cell reconstitution experiments, that SP–NK-1R signaling contributes to cardiac fibrosis not primarily through direct mast cell activation but rather through regulation of mast cell maturation, implying that the neuroimmune axis governs not only the acute responsiveness of cardiac mast cells but also the size and phenotypic composition of the resident mast cell pool (74). In a separate study in diabetic db/db mice, NK-1R agonism with GR73632 reduced fibrosis and improved diastolic function, indicating that the direction of benefit depends on the disease context (75).

The neuroimmune regulation of cardiac mast cells extends to direct anatomical and functional coupling between sensory C-fibers and perineuronal mast cells. Morrey et al. characterized this interaction in detail, demonstrating that capsaicin-mediated C-fiber activation triggered the release of SP and CGRP, which in turn induced mast cell degranulation and renin secretion, with the locally generated Ang II potentiating NE release from sympathetic nerve terminals and creating a self-amplifying neuroimmune–RAS loop that culminated in severe arrhythmic dysfunction (39). The pharmacological significance of this pathway was further contextualized by Reid et al., who reviewed evidence that cardiac mast cells are strategically positioned at perivascular and perineuronal sites to enable rapid modulation of local RAS activity, and that pharmacological stabilization of cardiac mast cells or inhibition of their renin output attenuates arrhythmic vulnerability in I/R models (40). The hypothalamic arm of the neuroimmune axis introduces an additional tier of regulation in which corticotropin-releasing hormone (CRH) activates coronary mast cells via CRHR-1 and CRHR-2, and this activation is amplified by co-stimulation with neurotensin and urocortin, with the resulting autocrine-paracrine CRH loop sustaining chronic coronary microvascular inflammation and contributing to the pathophysiology of HFpEF and mast cell activation disease (76). The therapeutic tractability of the neuroimmune–mast cell axis is powerfully illustrated by the cardioprotective effects of relaxin, which significantly reduced plasma histamine levels, increased cardiac histamine content, and decreased the extent of mast cell degranulation in a porcine cardiac I/R model, accompanied by reduced oxidative cardiac injury and a lower incidence of severe ventricular arrhythmias (23). The convergence of evidence from substance P antagonism (73), NK-1R–mediated mast cell maturation control (74), C-fiber–mast cell–local RAS coupling (39, 40), hypothalamic CRH–coronary mast cell signaling (76), and relaxin-mediated mast cell stabilization (23) collectively delineates a multi-nodal neuroimmune network in which cardiac mast cells function as integrative hubs that receive, amplify, and transduce diverse neural and hormonal stress signals into a coordinated program of myocardial injury, remodeling, and arrhythmogenesis.

## Metabolic and intracellular signaling mechanisms

Beyond their established roles as sources of vasoactive and fibrogenic mediators, cardiac mast cells are increasingly recognized as metabolically integrated effector cells whose degranulation and pathological activation are governed by discrete intracellular signaling cascades that intersect with mitochondrial function, oxidative stress, epigenetic reprogramming, and cell-autonomous cardioprotective pathways. Deshwal et al. established in streptozotocin-induced diabetic mice that monoamine oxidase (MAO)–dependent ROS generation constitutes the upstream trigger of a pathogenic cascade in which mitochondrial permeability transition pore (mPTP) opening and mitochondrial dysfunction precede endoplasmic reticulum (ER) stress, which in turn drives mast cell degranulation and abnormal collagen deposition culminating in diastolic dysfunction, with MAO inhibitor pargyline interrupting this MAO–ROS–mPTP–ER stress–mast cell axis and preventing degranulation and normalizing diastolic function (77). The ROS–mitochondrial–ER sequence was examined in cardiomyocytes; the intervention was not mast cell–specific. Palaniyandi et al. demonstrated in Dahl salt-sensitive rats subjected to high-salt diet–induced hypertensive heart failure that epsilon protein kinase C (ϵPKC) serves as a critical intracellular signal mediating pathological mast cell degranulation, as the selective ϵPKC inhibitor ϵV1–2 attenuated mast cell degranulation without altering mast cell density, fibrosis, or inflammation, demonstrating that the pathological output of cardiac mast cells in hypertensive heart failure is regulated at the level of intracellular signal transduction rather than cell number (78). Hans et al. demonstrated in *Apoe*-deficient mice fed a high-fat diet that PARP-1 drives a cascade of MMP-9 activation, reduced TIMP-2 expression, and mast cell degranulation that collectively precipitate cardiac hypertrophy and dilation, with genetic deletion of PARP-1 preventing cardiac hypertrophy and dilation and markedly attenuating mast cell degranulation (53). A fundamentally distinct epigenetic mechanism was identified by Zhou et al., who demonstrated that the ganglioside synthase *St8sia1* is selectively induced in cardiac mast cells during pathological remodeling, whereupon its product GD3 binds histone variants H2A.Z and H3.3C to reprogram chromatin accessibility and activate pro-inflammatory and pro-fibrotic transcriptional programs, with GD3 inhibition suppressing mast cell degranulation, disrupting pathogenic mast cell–cardiomyocyte and mast cell–fibroblast crosstalk, and preserving reparative macrophage populations (79). Here, “glyco-epigenetic regulation” refers to the reported link between ganglioside metabolism and chromatin regulation in mast cells. Its broader relevance across cardiac disease settings remains to be established.

In counterpoint to these pathological metabolic cascades, a series of mechanistically convergent studies has delineated a cell-autonomous cardioprotective metabolic pathway in cardiac mast cells centered on the PKCϵ–aldehyde dehydrogenase type-2 (ALDH2) axis. Koda et al. established that ischemic preconditioning (IPC) and adenosine A_2_b/A_3_ receptor activation trigger PKCϵ-mediated ALDH2 activation in cardiac mast cells, preventing renin release, local Ang II formation, norepinephrine overflow, and ventricular fibrillation, and identifying this PKCϵ/ALDH2 pathway as the critical anti-RAS cardioprotective mechanism of IPC (80). Aldi et al. demonstrated that histamine H_4_ receptor (H_4_R) activation on cardiac mast cells inhibits renin release through a PKCϵ-dependent ALDH2 activation mechanism, establishing H_4_R–PKCϵ–ALDH2 as a discrete cardioprotective signaling axis amenable to pharmacological exploitation (81). Marino and Levi synthesized mechanistic evidence demonstrating that Gi-coupled receptor activation, encompassing histamine H_4_R, adenosine A_3_R, and sphingosine-1-phosphate S1P_1_R, converges on sequential PKCϵ and mitochondrial ALDH2 activation in cardiac mast cells to prevent toxic aldehyde-induced renin release, thereby blocking local RAS activation and reperfusion arrhythmias, and positioning H_4_R, A_3_R, and S1P_1_R as a triad of pharmacologically accessible targets for ischemic heart disease (82). Taken together, the evidence reviewed in this section reveals that cardiac mast cell biology in heart failure and cardiomyopathy is inseparably intertwined with the metabolic state of the myocardium, with MAO-generated ROS in diabetes (77), ϵPKC-mediated degranulatory signaling in hypertension (78), PARP-1–driven MMP-9 activation in dyslipidemia (53), and GD3-mediated glyco-epigenetic transcriptional reprogramming in pressure overload (79) each representing disease-specific metabolic inputs that converge on mast cell activation as a common pathological output, while the PKCϵ–ALDH2 axis constitutes an endogenous metabolic brake on mast cell renin secretion whose pharmacological engagement offers a mechanistically grounded strategy for cardioprotection (80–82).

## Management of heart failure based on mast cell mechanisms

The mechanistic evidence reviewed in the preceding sections collectively identifies cardiac MCs as a therapeutically actionable cellular target across multiple heart failure etiologies, and supports investigation of mast cell–directed pharmacological strategies alongside existing neurohormonal pharmacotherapy; clinical superiority has not been established. The principal therapeutic approaches that emerge from this evidence base can be organized according to their primary molecular target within the mast cell effector cascade, as summarized in **Table 1**.

**Table 1:** Mast cell–targeted approaches and their supporting evidence.

Therapeutic target	Agent class/examples	Mechanism	Evidence level
Mast cell degranulation (broad stabilization)	Cromoglycate; nedocromil; ketotifen	Reduce granule mediator release	Preclinical: overload, I/R, and myocarditis models (18, 24, 29)
Chymase	TY-51469; SUNC8257; HA28; fulacimstat	Block Ang II generation, IGF-1 degradation, TGF-β activation, and ECM remodeling	Preclinical benefit; CHIARA MIA 2 showed no improvement in LV remodeling (37, 42, 43, 64, 65, 67, 83)
Histamine H_1_ receptor	Bepotastine; cetirizine	Reduce inflammatory injury and microvascular leakage	Preclinical: viral myocarditis and diabetic microvascular dysfunction (28, 84)
Histamine H_2_ receptor	Famotidine	H_2_ blockade; remodeling and symptom measures	Retrospective and small open-label randomized evidence; preliminary (85)
Histamine H_4_ receptor	H_4_R agonists	Activate the PKCϵ–ALDH2 pathway and limit renin release	Preclinical I/R evidence (81, 82)
Mast cell-derived renin	BILA2157; ALDH2 activators	Reduce local Ang II, NE overflow, and arrhythmias	Preclinical I/R evidence (6, 80)
NK-1R	L732138; GR73632	Context-dependent effects on mast cell maturation and remodeling	Preclinical: antagonist and agonist effects vary by model (73–75)
ϵPKC	ϵV1-2	Reduce degranulation; fibrosis and inflammation unchanged	Preclinical hypertensive HF evidence (78)
MAO/mitochondrial ROS	Pargyline	Interrupt the MAO–ROS–mPTP–ER stress cascade; not mast cell-specific	Preclinical diabetic cardiomyopathy evidence (77)
*St8sia1*/GD3 axis	Mast cell-specific *St8sia1* deletion; anti-GD3 R24	Suppress chromatin reprogramming and inflammatory activation	Preclinical pressure-overload and I/R evidence (79)
TSLP–mast cell axis	Genetic follow-up; TSLP overexpression	Strain-associated fibroblast–mast cell signaling and fibrosis	Mechanistic evidence; therapeutic benefit unestablished (52)
PDGFR-α	PDGFR-α neutralizing antibodies	Reduce mast cell-driven atrial fibrosis and AF inducibility	Preclinical pressure-overload AF evidence (35)

One strategy for clinical investigation is the repurposing of mast cell stabilizers, cromoglycate, nedocromil, and ketotifen, as adjunctive agents in heart failure management. Brower and Janicki demonstrated that nedocromil dose-dependently prevented left ventricular dilation and contractility decline in a rat aortocaval fistula model, significantly reducing congestive heart failure morbidity and mortality (24), while Mina et al. demonstrated that cromolyn sodium prevented left ventricular dilation, attenuated dysfunction, and reduced myocardial fibrosis in fulminant autoimmune myocarditis (29). These agents are already approved for allergic and asthmatic indications, possess well-characterized safety profiles, and could in principle be evaluated in randomized clinical trials as adjuncts to standard neurohormonal therapy in patients with inflammatory cardiomyopathy, volume overload heart failure, or HFpEF. The rationale for their use in HFpEF is particularly compelling given the evidence that mast cell–derived histamine drives coronary microvascular endothelial dysfunction and diastolic dysfunction in obese diabetic mice (84) and the proposed role of the mast cell–histamine–microvascular axis in HFpEF (86). However, established HF therapies already improve outcomes: DELIVER showed that dapagliflozin reduced the composite of worsening HF or cardiovascular death in patients with an ejection fraction above 40% (87).

Chymase inhibition represents the most mechanistically validated and pharmacologically advanced mast cell–targeted strategy for heart failure management. The evidence that chymase generates Ang II independently of ACE (42–44), degrades IGF-1 to promote cardiomyocyte death (64), activates TGF-β to drive fibrosis (37), and cleaves ECM components to precipitate ventricular dilation (48) establishes chymase as a pleiotropic mediator of adverse remodeling whose inhibition would be expected to provide benefits across multiple pathological axes simultaneously. The superiority of combined chymase and ACE inhibition over ACE inhibition alone in experimental animals in improving post-MI LV function and survival (42) provides a particularly strong rationale for clinical evaluation of chymase inhibitors as add-on therapy in patients with ischemic cardiomyopathy who remain symptomatic on standard RAAS blockade. The development of orally active chymase inhibitors (71, 72) and RNA aptamer–based inhibitors (67) provides a range of pharmacological modalities suitable for different clinical contexts, from acute post-MI settings to chronic heart failure management. However, in the randomized CHIARA MIA 2 trial involving 107 patients after myocardial infarction, fulacimstat did not improve changes in LVEF or ventricular volumes over six months compared with placebo (83).

Several factors may contribute to this divergence. ACE and cathepsin G can also generate Ang II (42, 69), so selective chymase inhibition may not suppress all local Ang II formation. Species differences also complicate extrapolation: unlike human chymase, mouse mast cell protease 4 has appreciable Ang II-degrading activity (42). Intervention timing differs as well. In a hamster MI model, treatment began 24 hours after coronary occlusion (42), whereas fulacimstat was started 6–12 days after MI in CHIARA MIA 2 and was given on top of standard care (83). These differences may affect the efficacy of chymase inhibition and contribute to the discrepancy between preclinical and clinical findings. Further studies are therefore needed to determine whether the favorable preclinical results translate into clinical benefit.

Antihistamine therapy targeting H_1_ and H_2_ receptors represents a mechanistically grounded candidate strategy whose efficacy as adjunctive heart failure treatment remains unestablished, particularly in inflammatory cardiomyopathy and HFpEF. The demonstration that bepotastine, an H_1_ antagonist, improved survival and reduced cardiac injury in viral myocarditis (28), and that H_1_ receptor activation cooperates with mast cell protease release to amplify myocardial damage, provides a mechanistic basis for clinical trials of antihistamines in patients with myocarditis and inflammatory cardiomyopathy. For H_2_ blockade, famotidine showed favorable findings in a retrospective study and a small open-label randomized study involving 50 patients with chronic HF; these findings remain preliminary (85). The H_4_ receptor agonist strategy, which activates the PKCϵ–ALDH2 cardioprotective axis within cardiac mast cells to prevent renin release and reperfusion arrhythmias (81, 82), represents a more novel and mechanistically precise approach that could be evaluated in the context of acute MI and I/R injury, where the mast cell–renin–Ang II cascade is most acutely activated.

The neuroimmune axis offers additional therapeutic opportunities through NK-1R antagonism and CRH receptor blockade. NK-1R antagonists, which are approved for chemotherapy-induced nausea and vomiting (e.g., aprepitant), could be repurposed for evaluation in volume overload heart failure and hypertensive cardiomyopathy, where experimental studies implicate SP–NK-1R signaling in remodeling and mast cell maturation (73, 74). This rationale cannot be generalized to diabetic cardiomyopathy, in which NK-1R agonism was protective (75). The hypothalamic CRH–coronary mast cell axis identified by Papadopoulou and Theoharides (76) suggests that psychological stress management and CRH receptor–targeted interventions may attenuate the neuroimmune component of HFpEF pathogenesis, a hypothesis that warrants prospective investigation given the well-documented association between psychological stress and HFpEF exacerbation. The identification of natural flavonoids as multi-target inhibitors of the CRH–mast cell pathway (76) provides a mechanistic rationale for dietary and nutraceutical interventions as candidates for investigation; the cited review does not establish clinical benefit in HFpEF.

The metabolic signaling mechanisms reviewed above suggest several additional therapeutic opportunities. MAO inhibition with pargyline or related compounds could attenuate mast cell–driven adverse remodeling in diabetic cardiomyopathy by interrupting the MAO–ROS–mPTP–ER stress–mast cell degranulation cascade (77). ϵPKC inhibition with peptide inhibitors such as ϵV1–2 could selectively suppress mast cell degranulatory activity in hypertensive heart failure without depleting the resident mast cell population (78), but preservation of reparative functions was not demonstrated, and fibrosis and inflammation were unchanged in the reported comparison. The *St8sia1*–GD3 glyco-epigenetic axis identified by Zhou et al. (79) represents a particularly promising therapeutic target because it operates at the level of chromatin reprogramming, with mast cell–specific *St8sia1* deletion and anti-GD3 antibody R24 providing experimental support; durable benefit from a single intervention has not been established. The TSLP–mast cell fibrogenic axis identified by Pimpalwar et al. (52) suggests that anti-TSLP biologics, already in clinical use for severe asthma (tezepelumab), could be repurposed for evaluation in pressure overload–induced cardiac fibrosis, although the cited study did not test tezepelumab as a cardiac treatment.

A critical consideration in the clinical development of mast cell–targeted therapies for heart failure is the preservation of the context-dependent protective functions of cardiac mast cells. The evidence that mast cell deficiency exacerbates cardiac remodeling under certain conditions (10), that mast cell tryptase maintains myofilament Ca^2+^ sensitivity in the post-ischemic myocardium (13), and that tryptase induces endothelial chemokines that may contribute to repair after MI (63) collectively argues against global mast cell depletion as a therapeutic strategy and in favor of selective targeting of specific pathological mediator pathways. A proposed therapeutic approach might combine mast cell stabilization to prevent acute degranulation in the context of ischemia and hemodynamic stress with selective inhibition of the most pathologically consequential mediators, chymase, histamine, and CXCL4, while preserving the tryptase-mediated reparative and cardioprotective functions of the resident mast cell population; broad stabilization has not been shown to achieve this selectivity. Achieving this therapeutic precision will require the development of subtype-selective mast cell modulators informed by the transcriptomic heterogeneity of cardiac mast cells across disease states, as characterized by Sandstedt et al. (88), and the integration of cardiac mast cell biomarkers, including serum chymase (68) and tissue mast cell density (7), into clinical trial design to enable patient stratification and pharmacodynamic monitoring of mast cell–targeted interventions.

## Discussion and future perspectives

Despite the substantial mechanistic insights accumulated over the past two decades, the translation of cardiac mast cell biology into effective clinical therapies for heart failure and cardiomyopathy remains at an early stage, and several critical questions must be resolved before mast cell–targeted interventions can be rationally designed and implemented. The most pressing frontier is the comprehensive characterization of human cardiac mast cell heterogeneity and transcriptomic reprogramming in the diseased heart. Sandstedt et al. recently provided the first in-depth transcriptomic characterization of mast cells isolated from failing human hearts, demonstrating through RNA sequencing that cardiac mast cells in end-stage heart failure exhibit significant activation of fibrosis-associated and inflammatory gene networks that are not observed in mast cells from nonfailing controls, showing altered transcriptional pathways in sorted C-kit^+^/CD45^+^ cells from nine HF patients and five nonfailing donors, without determining whether these changes cause or follow HF (88). This dataset provides a foundation for identifying disease-specific mast cell subpopulations and mediator programs that may serve as precision therapeutic targets, and it underscores the necessity of moving beyond murine models to directly interrogate human cardiac mast cell biology at single-cell resolution. The broader landscape of cardiac mast cell heterogeneity and plasticity across cardiovascular diseases was synthesized by Poto, Marone, Galli, and Varricchi, who highlighted the significant organ-specific and disease-context–dependent variation in human cardiac mast cell mediator profiles and receptor expression, and concluded that subtype-specific intervention strategies, rather than global mast cell depletion, will be required to exploit the therapeutic potential of this cell population without abrogating its context-dependent protective functions (4). An important emerging dimension of cardiac mast cell biology is the discovery of novel epigenetic and glyco-epigenetic regulatory mechanisms that govern mast cell effector function in the heart. Zhou et al. identified mast cell *St8sia1* as a glyco-epigenetic checkpoint that drives cardiac remodeling, demonstrating that this sialyltransferase enzyme regulates mast cell activation state and downstream fibrogenic signaling in a manner that is amenable to pharmacological targeting, thereby opening a new class of mast cell–directed interventions distinct from conventional degranulation inhibitors (79). Future research must systematically map the epigenetic and post-translational regulatory landscape of cardiac mast cells across disease stages to identify additional checkpoints of this type that can be selectively modulated.

The extension of cardiac mast cell pathobiology to previously undercharacterized disease contexts represents another major future direction. In the context of heart failure with preserved ejection fraction (HFpEF), Guimbal et al. demonstrated that cardiac mast cells are the trigger of small vessel disease and diastolic dysfunction in obese diabetic mice, with mast cell–derived histamine identified as the key mediator of endothelial dysfunction and coronary microvascular disease, and cromolyn sodium and the H_1_ antagonist cetirizine reducing microvascular leakage and diastolic dysfunction in female db/db mice (84). The clinical significance of this finding was contextualized by Bot, who highlighted the strategic perivascular localization of cardiac mast cells as the anatomical basis for their capacity to impair microvascular integrity and called for systematic investigation of mast cell contributions to coronary microvascular dysfunction as a mechanistic basis for HFpEF (86). Although therapies such as dapagliflozin improve HF outcomes (87), coronary microvascular dysfunction remains an important pathophysiological feature. The mast cell–histamine–microvascular axis identified by these studies warrants further translational investigation. The role of mast cell–fibroblast crosstalk in mediating myocardial fibrosis has been further illuminated by Pimpalwar et al., who demonstrated that TSLP mediates strain-induced fibroblast–mast cell crosstalk and fibrosis in the heart, implicating the TSLP–mast cell axis as a mechanistic link between hemodynamic stress and fibrogenic activation and identifying TSLP signaling as a potential therapeutic target for preventing pressure overload–induced cardiac fibrosis (52). In the emerging field of peripartum cardiomyopathy, Mulvey et al. identified mast cell chymase as a new diagnostic candidate through unbiased molecular characterization of peripartum cardiomyopathy hearts, suggesting that mast cell protease profiling may have diagnostic utility beyond its established mechanistic significance (68). The role of mast cells in hypertensive heart disease was further contextualized by Gouveia, who proposed that mast cell–derived TGF-β1, chymase, and bFGF are central drivers of the fibrotic remodeling characteristic of hypertensive cardiomyopathy and advocated for mast cell–targeted therapy as a complementary strategy alongside conventional antihypertensive treatment to prevent hypertension-induced diastolic dysfunction (89). Taken together, these converging lines of evidence from transcriptomics, epigenetics, novel disease models, and mechanistic pharmacology define a rich and clinically urgent research agenda for cardiac mast cell biology, centered on the need for human single-cell transcriptomic atlases of cardiac mast cells across disease states, the development of subtype-selective mast cell modulators that preserve protective functions while abrogating pathological mediator release, and the rigorous clinical evaluation of mast cell–targeted strategies in HFpEF, right heart failure, hypertensive cardiomyopathy, and peripartum cardiomyopathy, conditions that warrant further mechanistic and clinical investigation.

## Limitations

This review has several limitations. Much of the mechanistic evidence derives from animal or cell models, and its applicability to human heart failure remains uncertain. Kit-dependent mast cell-deficient models and pharmacological interventions may have effects beyond mast cells, limiting cell-specific causal inference. Human evidence is largely observational and does not establish causality. Randomized clinical evidence is limited for most mast cell-targeted interventions. As a narrative review, this article may also be subject to selection bias and does not provide a systematic assessment of all available evidence.
